# Depuration of geosmin- and 2-methylisoborneol-induced off-flavors in recirculating aquaculture system (RAS) farmed European whitefish *Coregonus lavaretus*

**DOI:** 10.1007/s13197-019-03910-7

**Published:** 2019-07-10

**Authors:** P. C. Lindholm-Lehto, J. Vielma, H. Pakkanen, R. Alén

**Affiliations:** 1 Aquatic Production Systems, Natural Resources Institute Finland (Luke), Survontie 9A, 40500 Jyväskylä, Finland; 2 Department of Chemistry, University of Jyväskylä, Box 35, 40014 Jyväskylä, Finland

**Keywords:** Depuration, European whitefish, Geosmin, 2-Methylisoborneol, RAS farming

## Abstract

European whitefish *Coregonus lavaretus* has increasingly become an important species for aquatic food production, especially in the Nordic countries. Whitefish is produced in traditional cage and pond operations, and in recirculating aquaculture system (RAS) in which, unfortunately, off-flavors and odors, mostly caused by geosmin (GSM) and 2-methylisoborneol (MIB), can accumulate in fish flesh from the circulating water. GSM and MIB have very low human sensory detection limits and, therefore, often disliked by consumers even at low concentrations. In this study, concentrations of GSM and MIB in RAS farmed European whitefish were studied by an analytical method based on headspace solid phase microextraction and gas chromatography–mass spectrometry. Concentrations were determined in different parts of fish: fillet, neck, belly, and tail during a depuration period and in depuration water. The highest initial concentrations were on average 32 ng g^−1^ (GSM) and 24 ng g^−1^ (MIB) in European whitefish fillet and 128 ng L^−1^ (GSM) and 94 ng L^−1^ (MIB) in water, respectively. After a depuration period of 16 days, concentrations decreased to below the detection limits, indicating the importance of the depuration period.

**Electronic supplementary material:**

The online version of this article (10.1007/s13197-019-03910-7) contains supplementary material, which is available to authorized users.

## Introduction

Aquaculture has been a rapidly growing food producing sector and nowadays accounts for more than half of the fish used for human consumption (FAO 2016). Off-flavor-induced tainting of fish has traditionally occurred in outdoor ponds but has often been observed also in recirculating systems (Houle et al. 2011; Schrader and Dennis 2005). In recirculating aquaculture systems (RAS), unwanted off-flavors with unpleasant odors can accumulate in circulating water and in fish flesh (Hathurusingha and Davey 2014; Houle et al. 2011; Howgate 2004). Off-flavors in fish flesh have been widely documented for, e.g. arctic charr *Salvelinus alpinus* (Houle et al. 2011), Atlantic salmon *Salmo salar* (Burr et al. 2012), barramundi *Lates calcarifer* (Percival et al. 2008), largemouth bass *Micropterus salmoides*, Nile tilapia *Oreochromis niloticus* (Yamprayoon and Noomhorm 2003), and rainbow trout *Oncorhynchus mykiss* (Robertson et al. 2005). The presence of off-flavor in fish flesh is perceived as a quality defect and often disliked by consumers, which can lead to high financial losses in producers’ earnings. Off-flavors are typically removed by depurating the fish in clean water. Unfortunately, this often takes from days to weeks, and can lead even to significant economic losses due to delays of harvest and high consumption of clean water (Burr et al. 2012).

Off-flavor is often caused by two saturated bicyclic terpenoids, geosmin (GSM, *trans*-1,10-dimethyl-*trans*-9-decalol) and 2-methylisoborneol (MIB (1-R-exo)-1,2,7,7-tetramethyl-bicyclo[2.2.1]heptan-2-ol) (Gerber 1968, 1969), producing earthy and musty odor and taste. GSM and MIB are produced as secondary metabolites by a variety of bacteria, including actinomycetes, cyanobacteria, proteobacteria and fungi (Dickschat et al. 2005). Typically, streptomyces, myxobacteria, and actinomycetes are the organisms responsible for GSM and MIB production in indoor recirculating systems (Dickschat et al. 2005). Water phase and the biofilters have been suggested as the main source of GSM-producers in RAS, but so far, there is no consensus on which RAS compartment is dominant in GSM and MIB production (Lukassen et al. 2017).

GSM and MIB are semi-volatile compounds with octanol-water partition coefficients 3.57 for GSM and 3.31 for MIB (Howgate 2004). Even low concentrations in water can be absorbed by fish and accumulated in lipid-rich tissues (Houle et al. 2011; Howgate 2004), giving an unpleasant taste and odor to water (Smith et al. 2002) and fish (Howgate 2004). Additionally, both compounds are perceived at very low concentrations (< 1 ng g^−1^) by human senses (Davidson et al. 2014; Robertson et al. 2005).

The fat content of fish has an effect on the flavor sensation and the sensory threshold of lipophilic GSM and MIB (Howgate 2004; Zimba and Grimm 2015). Sensory thresholds of GSM and MIB (muddy and earthy flavor) increase with fat contents of fish (Drake et al. 2010; Howgate 2004), leaving a higher concentration undetected as the fat content increases. Threshold values are used in sensory analyses and generally defined as the probability of detection (Ridgway et al. 2010). According to the definition, 50% of population will detect the taint at that level. However, individuals have different abilities in detecting tastes and odors and some individuals can be more sensitive to certain compounds.

GSM and MIB are absorbed into the fish via gills, skin and gastrointestinal tract by lipid-rich tissues, gills being the main path of uptake (Howgate 2004). GSM and MIB are concentrated into the fish flesh until an equilibrium state is reached between the water phase and the fish. Their concentrations in water and the time of exposure are the main factors affecting those in fish flesh (Howgate 2004), but also e.g. fish species, water temperature, size, and age of fish are of importance (Percival et al. 2008). The exchange of chemicals is assumed to proceed through a passive process, affected by the lipophilic nature of the compounds and the concentration in the aqueous part of fish (Howgate 2004).

Several methods have been studied and tested to remove or decrease the formation of GSM and MIB in RAS, including ozonation (Powell and Scolding 2018), other advanced oxidation processes (AOPs) (Rurangwa and Verdegem 2015), algicides (Hathurusingha and Davey 2014), adsorption, for example, with activated carbon (Burr et al. 2012), and zeolites (Ghasemi et al. 2018). More recently, methods based on photocatalysis, such as modified TiO_2_ with sun light (Fotiou et al. 2015) and palladium modified tungsten trioxide photocatalyst (Xue et al. 2016) to degrade off-flavor compounds seem promising in the future. However, depurating with clean water is so far the only efficient method available to remove the off-flavors. Depuration can take from days to weeks, depending on, for example, the initial concentrations of GSM and MIB, the species, and the size of fish, but requires large volumes of water (Burr et al. 2012). Additionally, fish are typically not fed during the depuration to ensure as good water quality and efficient depuration as possible, which can lead to their weight loss.

European whitefish *Coregonus lavaretus* is a white-fleshed freshwater species which belongs to the family *Salmonidae* and a genus *Coregonus* spp. The group includes marine, anadromous, and freshwater species widely distributed in Northern Europe, North America, and Asia (Boiteanu et al. 2016). In recent years, European whitefish has increasingly become an emerging species for aquacultural food production in the Nordic countries. Typically, whitefish is consumed as raw pickled, smoked, fillet or other products, including caviar (Setälä 2008).

So far, concentrations in of GSM and MIB in fish flesh of different species have been a subject of limited number of studies. Additionally, only few studies have been conducted to detect the location of off-flavor accumulation in fish (Zimba and Grimm 2015). According to our knowledge, this is the first study regarding GSM and MIB concentrations in European whitefish during a depuration period. Aim of this research was to extensively study the location of off-flavor accumulation in fish flesh and the time required for sufficient depuration.

## Materials and methods

### Chemicals and materials

Standard solution (TraceCERT^®^, 100 µg mL^−1^ in MeOH) of (+/−) GSM and MIB were purchased from Merck. High-performance liquid chromatography (HPLC) grade methanol and hexane were obtained from J.T Baker and solid NaCl (purity 98%) from Merck. Ultra-high quality (UHQ) water from Millipore (Bedford, MA, USA) was used in the analyses. Additionally, 10 mL headspace (HS) glass vials and polytetrafluoroethylene (PTFE) septum caps were purchased from Merck.

Manual solid phase microextraction (SPME) assembly with an extraction fiber coated with StableFlex divinylbenzene/carboxene/polydimethyl siloxane (DVB/CAR/PDMS), 1 cm, 50/30 µm (part no. 57328-U) in a manual holder were purchased from Supelco (Merck).

### Sampling and pretreatment

#### Process conditions in RAS

European whitefish was raised in a commercial indoor RAS farm. The fish were fed until the beginning of the depuration period with commercially produced dried pellets, containing 0.8–1.7% phosphorous and 5.9–8.5% nitrogen with higher concentrations in juvenile feeds. In general, the main ingredients of feeds are fish meal, fish oil, soya, wheat, and rapeseed oil, supplemented with vitamins and trace elements. At the farm, average biomass of European whitefish was 30 t, fed 200 kg feed per day. In the RAS process, ammonium was removed by nitrification in the bio-filter, while dissolved carbon dioxide was removed from the water by packed aeration tower. In the circulation system, river water or water from a drill well was used as the source for the renewal water. The circulation water was disinfected with ozone and UV light, pH adjusted with sodium hydroxide or sodium bicarbonate, and aerated before circulating back to tanks. Solid organic material was removed with a drum filter and phosphorous material was precipitated by using polyaluminum chloride and polymer, leaving 2.5% of total phosphorous. The flocculated material was led to a band filter to remove the remaining water.

In the beginning of the depuration, the tank of 127 m^3^ with 5500 kg of fish filled with circulation water was put on flow through. River water was led to the depuration tank (3 L s^−1^), displacing the circulation water over time. Temperature of the incoming water remained unadjusted, being near 0 °C in the winter. The fishes were not fed during the depuration. Fish were sampled from one depuration tank during one procedure.

#### Sampling of fish and circulation water

Sampling of RAS farmed European whitefish was performed after 1, 3, 7, 10, 13 and 16 days of depuration. Five fishes were taken for each sampling. The fish were instantly frozen after sampling and stored at − 24 °C until melted and used for the analyses. On average, each fish weighted 600 g with a slaughter yield of 90.1%. De-frozen fishes were filleted and pieces (15 g ± 1 g) of neck, tail, stomach, and fillet as described by Hathurusingha and Davey (2016) were collected for analysis, consisting of 5 fish with four sampling points per fish. Four parallel analyses were performed for each sample piece (n = 5 × 4). All analyzed parts of fish consisted of flesh, while visible pieces of non-edible parts were discarded.

Water from the depuration tank was taken for analysis after 1, 2, 5 and 8 days. Water samples were collected in 1 L high-density polyethylene (HDPE) bottles and stored frozen at − 24 °C before the analyses. Parallel analyses (n = 4) were performed for each sample.

#### Lipid contents

Lipid contents of each sample piece (neck, tail, belly, and fillet) of fish after 1, 3, 7, 10, 13 and 16 days of depuration were determined by Synlab (Synlab, Analytics and Services Finland Oy). The accredited method (Synlab internal method 076) included lyophilization combined with acid hydrolysis to determine the lipid content of the fish flesh.

#### Pretreatment

GSM and MIB were extracted from the sample matrix by headspace solid phase microextraction (HS-SPME), modified from the method reported by Peng et al. (2014). First, 1 g of fish flesh or 1 mL of water was placed in a 10 mL HS vial. A saturated NaCl (aq) solution was prepared and 750 µL added to the vial. The method of standard addition with five additions was used to construct calibration curves for quantification of GSM and MIB in both fish and water samples. Four replicates of each sample were prepared and analyzed to determine the standard deviations.

Sealed sample vials were placed in a water bath at 60 °C. The septum of the sample vial was pierced with a needle and the DVB/CAR/PDMS fiber was exposed in the headspace for extraction. The extraction took 30 min before introducing the fiber directly into the GC–MS injection port for desorption.

### Gas chromatography–mass spectrometry (GC–MS)

An Agilent 6890 series/5973N GC/MSD (Palo Alto, CA, USA) system with a Phenomenex Zebron ZB-5MSi (Torrance, CA, USA) capillary column (30 m × 0.25 mm × 0.25 µm) was used to separate and detect the analytes. The temperature of the injector was adjusted to 270 °C in the splitless mode. The carrier gas was helium at the flow rate of 0.7 mL min^−1^. The temperature of the oven started at 45 °C for 3 min and increased 30 °C min^−1^ to achieve 300 °C (total time 14.5 min). The electron impact (EI)-MS conditions were selected 230 °C for ion source, 5 min delay time and ionizing voltage of 70 eV. Selected ion monitoring (SIM) mode was used for the detection of GSM and MIB with *m/z* 112, 126, 182 (GSM) and *m/z* 95, 135, 168 (MIB). Base peak areas of *m/z* 95 and 112 were used for the quantification of GSM and MIB.

### Method validation

The analytical method was validated in terms of linearity, level of detection (LOD), level of quantification (LOQ), sensitivity, precision, and repeatability. Method validation was determined in terms of circulation water and fish samples.

#### Linearity

Linearity was studied by five standard additions of GSM and MIB solutions to circulation water, ranging 10–50 ng L^−1^ and 20–60 ng g^−1^ to fish flesh. Both sample types showed adequate correlation coefficients and linearity: R^2^ 0.9877 (GSM) and R^2^ 0.9878 (MIB) for circulation water and R^2^ 0.9862 (GSM) and R^2^ 0.9883 (MIB) for fish flesh.

#### Sensitivity, levels of detection, and quantification

For the chromatographic analysis of GSM and MIB, LOD and LOQ were determined based on signal-to-noise ratio (S/N) = 3 for LOD and S/N = 10 for LOQ in the SIM mode. The results for water and fish samples are listed in Table 1. Additionally, sensitivities were calculated and also listed in Table 1.

**Table 1:** Level of detection (LOD), level of quantification (LOQ), and sensitivity of European whitefish (ng g^−1^), and circulation water samples (ng L^−1^)

Sample type	Compound	LOD	LOQ	Sensitivity
European whitefish *Coregonus lavaretus*	GSM, ng g^−1^	1.4	2.1	5.5
	MIB, ng g^−1^	0.5	1.5	0.9
Circulation water	GSM, ng L^−1^	2.1	2.8	2.3
	MIB, ng L^−1^	0.8	1.6	1.2

GSM—Geosmin, MIB—2-methylisoborneol

#### Precision and repeatability

Yields of standard addition of GSM and MIB standards are listed in Table 2. Yields have been calculated both for circulation water and whitefish flesh. Additions have been conducted with five replicative measurements (n = 5) and based on them, calculated the standard deviations (SDs).

**Table 2:** Yields of standard addition of GSM and MIB for circulation water (10–50 ng L^−1^ addition) and for European whitefish flesh (20–60 ng g^−1^ addition) with relative standard deviations (± RSD)

GSM (in water)		MIB (in water)		GSM (in whitefish)		MIB (in whitefish)	
Standard addition (ng L^−1^)	Yield (%)	Standard addition (ng L^−1^)	Yield (%)	Standard addition (ng g^−1^)	Yield (%)	Standard addition (ng g^−1^)	Yield (%)
10	94.5 ± 2.8	10	96.0 ± 2.6	20	101.1 ± 3.5	20	101.7 ± 3.6
20	94.1 ± 2.7	20	105.7 ± 2.1	30	97.6 ± 2.9	30	103.2 ± 3.0
30	99.4 ± 2.9	30	103.1 ± 1.3	40	96.3 ± 3.1	40	100.1 ± 3.2
40	96.9 ± 3.5	40	104.8 ± 2.8	50	103.8 ± 3.4	50	105.6 ± 2.9
50	98.6 ± 2.0	50	95.6 ± 2.2	60	103.7 ± 3.0	60	103.2 ± 3.3

GSM—Geosmin, MIB—2-methylisoborneol

### Depuration model

Modelling the depuration of European whitefish in general and the decrease of GSM and MIB in RAS system was performed as reported by Schram et al. (2017). The model was prepared to show the decline of GSM and MIB in relation to e.g. fish biomass, water flow rate, tank volume, and levels in the water. Based on this, the elimination rate constants were determined for European whitefish, which are generic, but still specific for the size, lipid content and temperature. Equation 1 describes the decrease of off-flavor concentrations in fish in clean water, assuming negligible accumulation of chemicals in the water.

1 $$ C_{B(t)} = C_{B(t = 0)} e^{{( - k_{2} t)}} $$

C_B(t = 0)_ is the initial concentration of GSM or MIB in fish in the beginning of the depuration period, t the time of depuration in hours, k_2_ elimination rate constant of GSM or MIB to the water, and C_B(t)_ the concentration of GSM or MIB in fish as a function of time.

Rate constant k_2_ is calculated for each body part (fillet, neck, belly, and tail) by the non-linear regression analysis based on the decrease of concentrations and the depuration time by Systat 13.1 (SYSTAT Software, Inc. 2009). By inserting the k_2_ values in Eq. 1 and recalculating per dry lipid contents, ln-transformed concentrations were obtained for each body part.

## Results and discussion

### GSM and MIB in whitefish

**Fig. 1:**
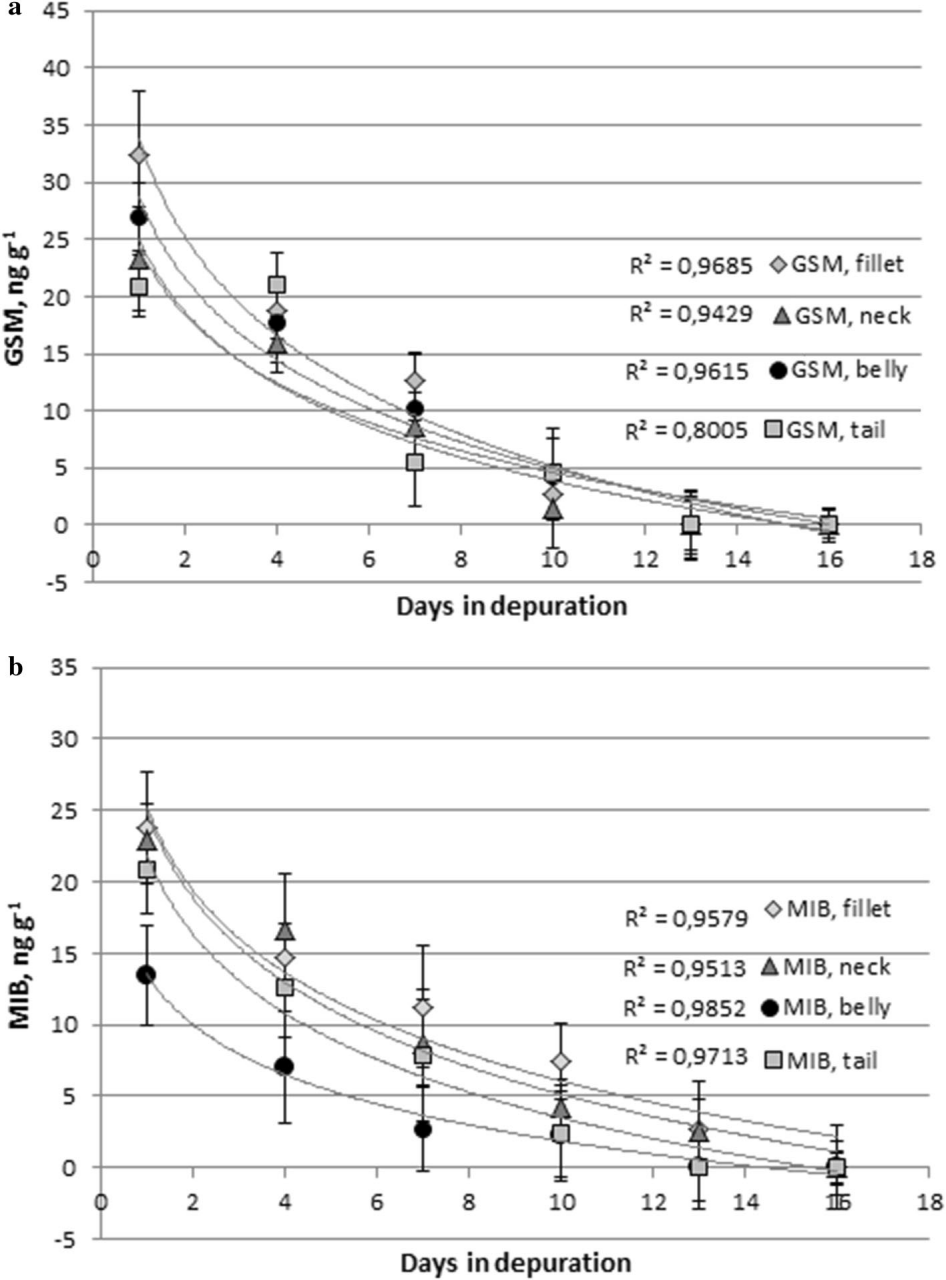
Concentrations of GSM (**a**) and MIB (**b**) in fillet, neck, belly, and tail of European whitefish flesh during the depuration of 16 days (± SD)

Concentrations of GSM and MIB in European whitefish fillet ranged from 0 to 32 ng g^−1^, decreasing during the 16 days of depuration (Fig. 1). Before the depuration period, concentrations were fairly high compared to 0.2–0.9 ng g^−1^ of MIB and 0.10–0.115 ng g^−1^ of GSM in Atlantic salmon (*S. salar*) reported by Davidson et al. (2014). Hathurusingha and Davey (2016) studied barramundi (*L. calcarifer*) in a circulation system with 6% (w/w) body fat and found 0.01–7 ng g^−1^ GSM and 0.01–7 ng g^−1^ MIB. On the other hand, higher concentrations of GSM and MIB have been reported by Zimba et al. (2012). They found 4.8–19.7 ng g^−1^ MIB and 0.27–0.59 ng g^−1^ GSM in RAS farmed rainbow trout (*O. mykiss*). Schram et al. (2017) studied depuration of European eel (*Anguilla anguilla*) with 33.7% (w/w) fat content and found high concentrations of GSM (22 ng g^−1^) before the depuration. The results were in the same range with those of this study.

The overall fat content of European whitefish with average size of 600 g was relatively high, but decreased over the time of depuration (Table 3). However, it is in the same range with that (11.1%) reported by Boiteanu et al. (2016) for farmed *C. maraena*, weighting 290–380 g. Kause et al. (2011) found 12.2% lipid content in European whitefish, ranging in size 540–710 g, while Airaksinen and Riihimäki (2014) reported lipid contents of about 9% for farmed whitefish *C. lavaretus* with harvest weight of 995 g. The lipid content after 10 days in depuration show sudden increase in all body parts of fish, although the lipid content in total shows up to 50% decrease during the time of depuration. This can be due to variation in fish size at different sampling points, supported by the increased limits of error at that point.

**Table 3:** Lipid contents (w/w, ± limit of error, %) of European whitefish *Coregonus lavaretus* after 1, 3, 7, 10, 13 and 16 days of depuration

Depuration time, days	Lipid content per dry flesh (%)			
	Neck	Fillet	Belly	Tail
1	24.9 ± 2.5	15.4 ± 1.5	30.1 ± 3.0	19.5 ± 2.0
3	18.0 ± 1.8	11.7 ± 1.1	15.6 ± 1.6	15.6 ± 1.6
7	17.5 ± 1.8	11.4 ± 1.1	17.0 ± 1.7	10.1 ± 1.0
10	21.2 ± 2.1	13.8 ± 1.4	22.7 ± 2.3	12.2 ± 1.2
13	13.0 ± 1.3	9.8 ± 1.0	17.3 ± 1.7	9.3 ± 1.9
16	12.8 ± 1.3	9.4 ± 1.9	13.3 ± 1.3	9.7 ± 1.0

The differences in fat contents were partly explained by the size of fish. However, other factors also have an effect on the fat content. Suomela et al. (2017) showed that the type of feed has a strong influence on the total lipid content of European whitefish and on the fatty acid profile of fish. Additionally, culture conditions affect the fatty acid composition as well as water temperature, becoming more unsaturated in colder water (Haard 1992).

The highest concentrations of GSM were detected in fillet and belly and of MIB in fillet and neck. In all parts of fish, concentrations decreased over the depuration period (Fig. 1). So far, there are only few studies available regarding off-flavors and fat contents in different parts of fish. Hathurusingha and Davey (2016) found the highest lipid content in the belly and the lowest in the tail part of barramundi. However, they did not determine concentrations of off-flavors in different parts of fish. Percival et al. (2008) stated that GSM and MIB were not homogeneously distributed in the fish but based on lipid concentrations, while Petersen et al. (2011) found no correlation between fat content and concentrations of GSM and MIB, only a positive correlation with the fish size. Percival et al. (2008) also stated that higher levels detected in larger fish is most likely caused by the higher lipid content of larger fish. In this study, the results indicated lower concentrations of MIB in the belly, the part with typically higher lipid contents compared to fillet and tail. In the case of GSM, the highest concentrations were detected in fillet and belly, and somewhat lower in the neck and tail. However, there were no significant differences between the average values of different body parts. It is possible, that GSM and MIB as lipophilic compounds were mostly concentrated in the fatty tissue. Only the edible flesh parts were studied and the fatty parts discarded which might have led to lower detected concentrations. On the other hand, this could be due to different species of fish and larger size (Percival et al. 2008).

The concentrations of GSM were mostly below the detection limits after 13 days of depuration (Fig. 1), while in the study of Robertson et al. (2005), the concentrations decreased rapidly at first (24 h) and then slower during the rest of the total 168 h of depuration. In the case of GSM, however, there is a more pronounced decrease in concentrations after the first 7 days (Fig. 1). On the other hand, Davidson et al. (2014) reported a slow decrease in MIB concentration during the first 6 days and an increased rate of decrease towards 10 days of depuration. The depuration performance and time of depuration depend on several factors, such as initial off-flavor concentration in fish, water temperature, fish density, water renewal rate, and the off-flavor concentration in the depuration system supply water (Drake et al. 2010; Howgate 2004), leading to varying rates of depuration.

The lowest initial levels of MIB were found in the belly, even though it contained the highest amount of fat. This may be explained by the fact that the lipophilic GSM and MIB find their ways especially into the fat tissue, while both flesh and fat were chosen for the chemical analysis. However, the rate of removal in the belly was similar to those in fillet, neck, and tail.

The rate constant k_2_ for the decrease of MIB was 0.007 h^−1^ for neck, 0.007 h^−1^ for belly, 0.005 h^−1^ for fillet, and 0.008 h^−1^ for tail. In the case of GSM, the rate constant k_2_ was 0.008 h^−1^ for neck, 0.008 h^−1^ for belly, 0.008 h^−1^ for fillet, and 0.007 h^−1^ for tail. These were somewhat lower than the rate constant k_2_ 0.014 h^−1^ reported by Schram et al. (2017), describing more efficient depuration. This is consistent with the slow change of circulating water to clean water in the beginning of the depuration.

**Fig. 2:**
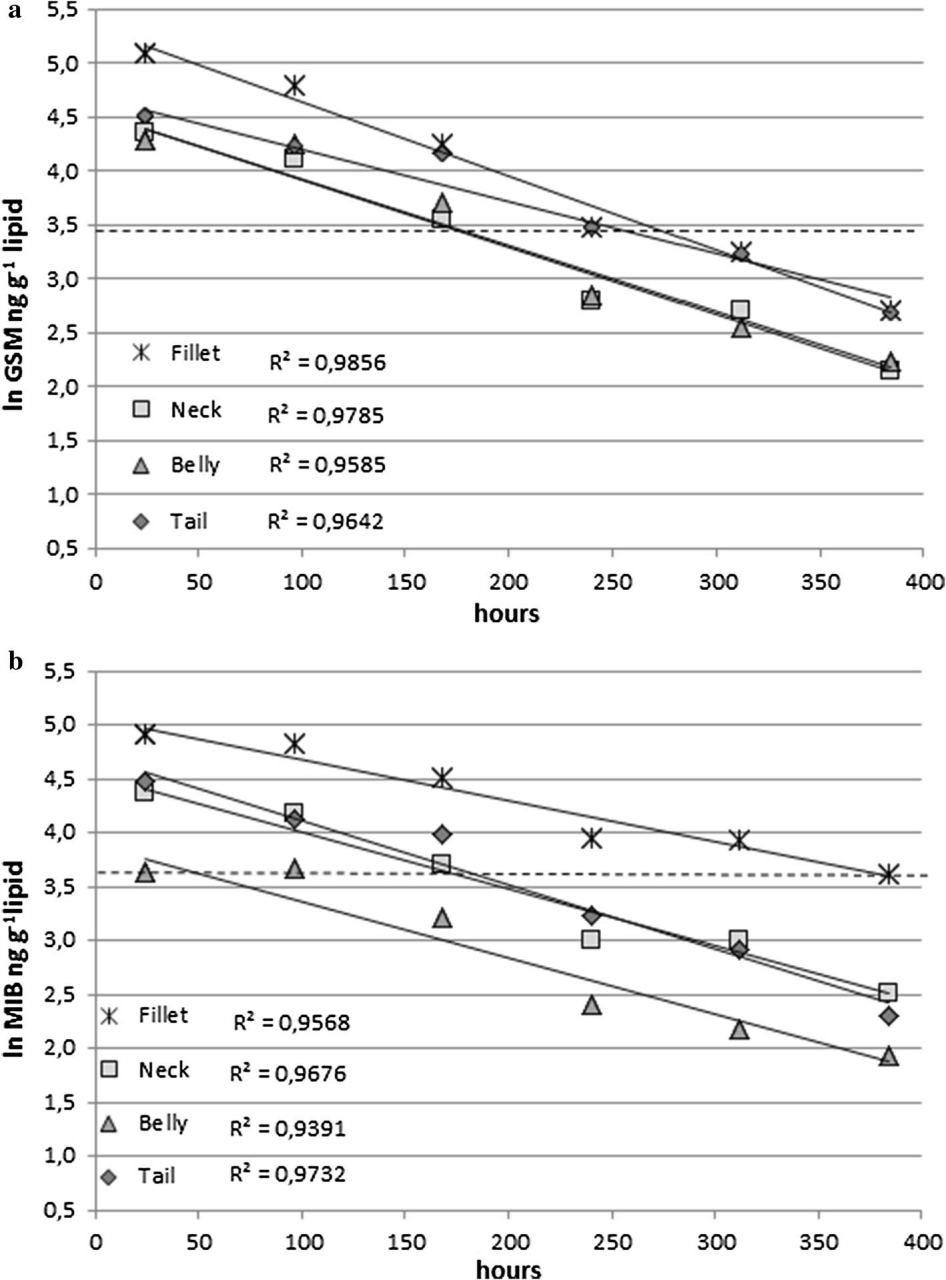
Ln transformed GSM (**a**) and MIB (**b**) concentrations in fillet, neck, belly, and tail of European whitefish (ng g^−1^ lipid) as a function of depuration time, 1–16 days (h). Dashed line describes the concentration of detection limits of GSM or MIB based on sensory and analytical methods

The decrease of GSM and MIB concentrations over the time of depuration for each body part has been presented Fig. 2 (ln GSM ng g^−1^ lipid). First, the rate constant k_2_ was calculated for fillet, neck, belly, and tail for both GSM and MIB (Supplementary file S1), which were used in recalculating the decease per lipid content. The rate of removal in different body parts proceeds at similar rates. The initial concentrations in fillet were higher than in the other body parts, despite the fact that the highest lipid contents were initially found in the belly and neck (Table 3). The results also show that even after 16 days of depuration, in some cases, low levels of off-flavor compounds remained in the fish flesh.

### GSM and MIB in water

**Fig. 3:**
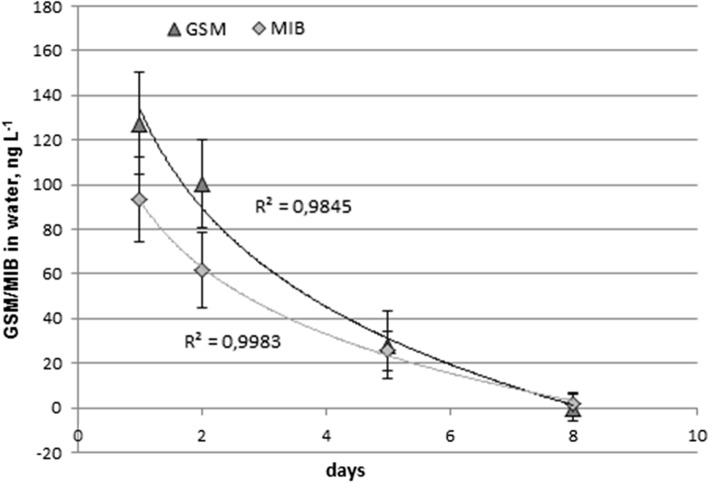
Concentrations of GSM and MIB in depuration tank during 1, 2, 5 and 8 days, n = 4 (± SD)

Concentrations in water decreased during the depuration time, as shown in Fig. 3. This is in agreement with the fact that at this particular farm, the circulation water was gradually changed to clean water during the time of depuration. In the beginning, the concentrations were fairly high, on average 128 ng L^−1^ for GSM and 94 ng L^−1^ for MIB. The results can be considered typical for circulation water of a RAS farm, even though the concentrations vary widely among RAS farms based on operational choices, process conditions, and water quality (Hathurusingha and Davey 2016).

GSM and MIB are assumed to be exchanged between water and fish by passive diffusion via the gills. The depuration process, driven by the fugacity and chemical potential, seeks equilibrium between the lipid fractions of fish and water, reaching equilibrium as the fugacities in lipids and water are equal (Howgate 2004). Even though GSM and MIB were excreted from the fish during the depuration period, the volume of water is very large compared to increase in GSM and MIB levels and typically, no net increase in their concentrations could be detected (Schram et al. 2017). However, very high fish densities and low water exchange rates can lead to increased levels of compounds excreted by the fish (Schram et al. 2009). In this study, the fish load during the depuration was at about 43 kg m^−3^. The results showed a decrease in concentrations of GSM and MIB both in fish and water (Figs. 1 and 3), suggesting an adequate volume of pure water for the depuration procedure. The concentrations detected in the depuration water mostly originated from the circulation water used to fill the depuration tank at the beginning of the depuration period, and GSM and MIB released by the fish. Additionally, biotransformation of GSM and MIB to their degradation products might occur (Schram et al. 2018).

## Conclusion

This study is the first to report GSM and MIB concentrations in European whitefish flesh and their distribution in different parts of fish. The levels found in European whitefish were likely caused by the high levels in circulation water and accumulation into the fish. In the future, studies regarding the accumulation in different species under similar conditions can give more insight.

The general depuration model was built to predict the removal of off-flavors from European whitefish more generally, with the effects of process conditions of this particular plant in minor part. The results show the great effect of fat content in European whitefish flesh. The decrease proceeds fairly similarly in all body parts, even though the initial concentrations were different. Additionally, the fat contents decreased up to 50% from their original values during the depuration, showing the effect of fasting.

The results show that depuration up to 16 days is required for MIB, but for GSM shorter time seems sufficient. In this study, circulation water was gradually changed to fresh water during the depuration period, increasing the time required for full removal of off-flavors. Optimization of the depuration time is crucial in order to reduce the production costs but still produce the high quality fish products. The importance of depuration is emphasized particularly, when producing mild-flavored European whitefish which must not provide any off-flavor sensation, not even for the most sensitive consumer.

## Electronic supplementary material

Below is the link to the electronic supplementary material.

Supplementary material 1 (DOCX 60 kb)
