# Supplementary material for: Depuration of geosmin- and 2-methylisoborneol-induced off-flavors in recirculating aquaculture system (RAS) farmed European whitefish Coregonus lavaretus
Source: J Food Sci Technol. 2019 Jul 10;56(10):4585–94. doi: 10.1007/s13197-019-03910-7 (PMC6801251; doi:10.1007/s13197-019-03910-7)
Supplement: Supplementary file 1 — Supplementary material 1 (DOCX 60 kb) [file 13197_2019_3910_MOESM1_ESM.docx]

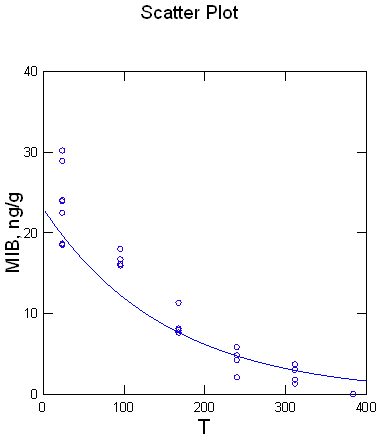

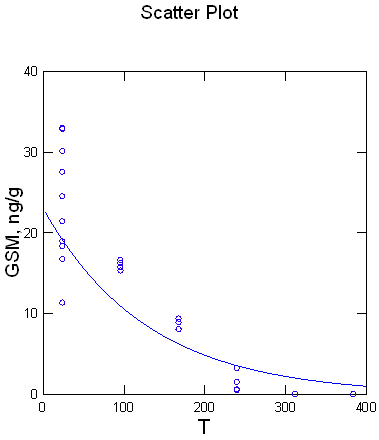


MIB, neck k_2_ 0.007 h^-1^, R^2^ 0.916 GSM, neck k_2_ 0.008 h^-1^, R^2^ 0.855


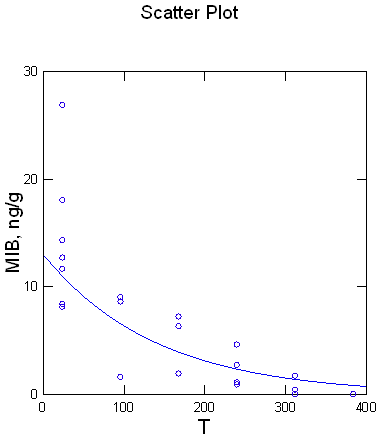

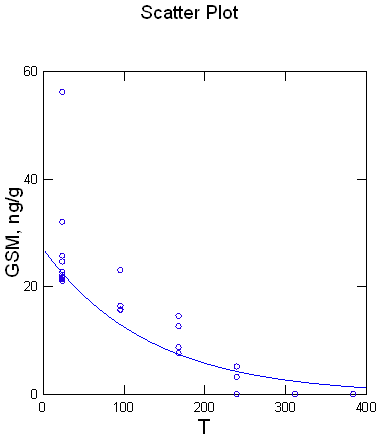


MIB, belly, k_2_ 0.007 h^-1^, R^2^ 0.779 GSM, belly, k_2_ 0.008 h^-1^, R^2^ 0.824


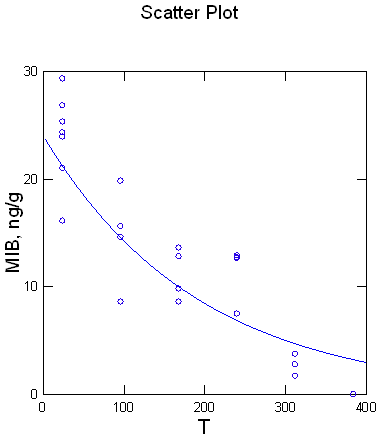

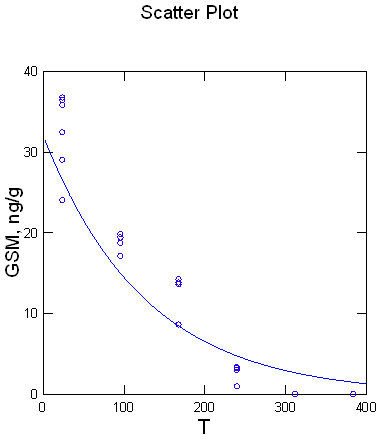


MIB, fillet, k_2_ 0.005 h^-1^, R^2^ 0.856 GSM, fillet k_2_ 0.008 h^-1^, R^2^ 0.884


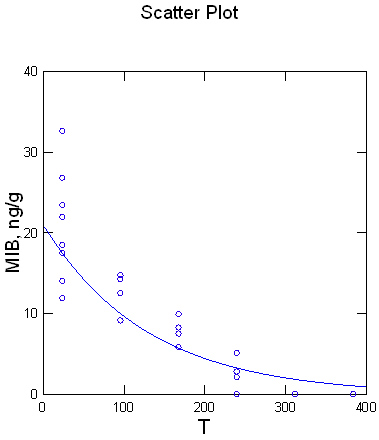

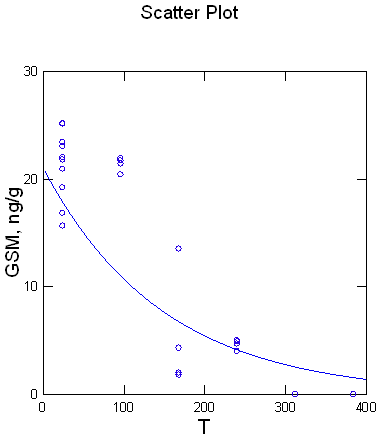


MIB, tail, k_2_ 0.008 h^-1^, R^2^ 0.860 GSM, tail, k_2_ 0.007 h^-1^, R^2^ 0.784

Supplementary Table S1. Constant k_2_ showing the rate of decrease of GSM and MIB in each body part (neck, belly, fillet, and tail) of whitefish *Coregonus lavaretus*.
